# Impacts of self- and cross-sucking on cattle health and performance

**DOI:** 10.14202/vetworld.2016.922-928

**Published:** 2016-09-01

**Authors:** Motamed Elsayed Mahmoud, Fatma Ali Mahmoud, Adel Elsayed Ahmed

**Affiliations:** 1 Department of Animal Behavior and Husbandry, Faculty of Veterinary Medicine, Sohag University, 82524 Sohag, Egypt; 2 Department of Animal Medicine, Faculty of Veterinary Medicine, South Valley University, Qena, Egypt

**Keywords:** animal welfare, culling, inter-sucking in cows, self-sucking in calves, teat deformity

## Abstract

**Background:**

Improvement of dairy farms economics requires intensification, automatic milking, and artificial rearing methods. The ability to express normal behavior is one of the five freedoms to achieve animal welfare, whereas the display of abnormal behaviors is considered as an indicator of poor welfare. Cross-sucking is defined as sucking any body parts of pen-mate calves, whereas inter-sucking in cows is defined as sucking the udder or udder area. Previous studies showed that self- and cross-sucking during the calf-hood period could be a causal factor of milk sucking in adulthood.

**Aim:**

To investigate the effects of cross-sucking among calves and inter-sucking in cows on animal health status and performance.

**Materials and Methods:**

Gathering information from customized questionnaires, the study of the breeding records, recording of self- and cross-sucking behaviors, and health status of calves till weaning, and dairy cows before milking were performed in two governmental farms under the same managemental conditions in Sohag and Qena governorates.

**Results:**

Cross-sucking appeared in calves at the 2^nd^ week of age followed by abscesses at ears and navels that were observed within cross-sucker calves. Milk sucking was higher in primiparous than multiparous cows during the second lactation period, as primiparous cows start to suck mostly around the 4^th^ month of milking. Mastitis and elongation of the front teats were observed in sucker cows. Suffered animals had body condition scoring 3.5 or less. Interestingly, most of the cows displaying self-sucking were sucking another cow and were experienced self- or cross-sucking in their calf-hood. The use of pronged nose-rings was ineffective in preventing milk sucking and all cows were ultimately culled at the end of the season.

**Conclusion:**

The results of this study demonstrate the health problems of abnormal oral behaviors in terms of developed ears and navels abscesses in cross-sucker calves, and mastitis and teat deformities in milk-sucker cows. Furthermore, indexes that lead to oral satisfaction should be taken in priorities of farm managers to effectively reduce or prevent cross-sucking in calves. Culling of cows and heifers suffering from sucking would be the ultimate uneconomic alternative in case of persistent suckers.

## Introduction

In modern dairy farms, calves are usually separated from their dams’ directly after birth and further social contact between them is usually prevented. Under natural condition, the cow-calf bonding develops soon after birth and usually persists for at least 1 year. It is recently indicated that keeping calves together with dam can develop cow-calf bond even without nursing [1]. Natural suckling is an important behavior for both mother and offspring. It allows transfer of milk from the dam to the young by displaying the act of sucking. In case of artificial rearing conditions, calves are mostly fed via bucket and sometimes by artificial teat to exhibit natural sucking behavior [2]. The artificial rearing of animals represents a combination of emotional (separation from the dam) and nutritional (transition from maternal milk to a commercial milk substitute) stressors in different species [3,4]. It has been suggested that rearing in contact with the mother during the first 12 weeks, even if very limited, may have a positive effect on behavior of heifer when introduced into the dairy herd, as various non-nutritive abnormal oral activities, including self-grooming and tongue playing and cross-sucking have been found to occur in calves fed with a bucket or reared in individual pen [5-7].

The majority of artificially reared calves develops abnormal oral behavior in terms of manipulating substrates or a pen-mate and tongue rolling [8,9]. However, such abnormal behaviors are rarely observed in naturally reared calves [10]. Milk sucking from the udder of heifers or cows is a frequent problem in dairy herds and may lead to udder damage, mastitis, milk loss, and culling of breeding animals. Such inter-sucking in cows was suggested to be a continuation of a habit that was already established in a calf-hood period [8].

Several strategies have been applied to minimize cross-sucking when calves are artificially fed [11,12]. Calves usually receive a restricted amount of whole milk or milk substitute (2-3 lb./meal/calf). The milk is usually consumed within 1 min, and thereafter, calves start sucking each other on different parts of the body, a behavior described as cross-sucking. This abnormal sucking is most intense during the first 6 min and then declines till 15 min after the milk ingestion when it stops [13]. Cross-sucking is considered a redirected natural sucking behavior toward peers, where calves started to suck each other, and the use of accessories that facilitate natural sucking behavior including teat-buckets, simple teat-feeders, and computer-controlled milk feeders could significantly reduce cross-sucking among calves [14,15]. Weaned heifers should have access to a ration which fulfills their energetic and behavioral needs to ensure optimal transition from pre-ruminant calves to ruminants. Hence, cross-sucking among calves is a behavior initiated and developed during the period of calf rearing, and its expression is reduced significantly by the offer of an enriched environment [16].

Artificial milk feeders are not efficient in completely satisfying sucking motivation [9,10]. Hence, the abnormal oral behaviors exhibited as a result of artificial rearing can be a sign of mental suffering due to unsatisfied animal’s needs [9,17]. Furthermore, artificial suckling contributes to the higher incidence of illnesses such as pneumonia and enteritis [10,11]. However, the hypothesis that development of diseases is related with cross-sucking should be further clarified. It has been reported that dairy calves are usually removed from the dam shortly after birth and raised in single or group pens during the milking period. On other hand, although restricted natural suckling could decrease abnormal sucking in calves and improve udder health in cows [13], different ways of offering milk to calves are used such as the provision of restricted amounts of whole milk or milk substitute in open buckets to the calves twice a day. Under natural conditions, the weaning process of calves is completed at 9-11 months of age [11,18]. However, in modern dairy farming, calves are weaned as soon as possible mainly for economic purposes, e.g., solid feed costs are less than that of milk, and the provision of milk demands costs much more labor than solid feed.

The aim of the present study was to investigate the effect of cross-sucking in calves on health status and body weight at weaning, and the development of inter-sucking in cows and its effects on udder health and behavior of cows before and during milking.

## Materials and Methods

### Ethical approval

The present study was approved by the Institutional Animal Ethics Committee of The Faculty of Veterinary Medicine at Sohag University.

### Animals and management

The study was performed on Holstein-Friesian cattle in two governmental farms; one in Sohag governorate and another in Qena governorate, thus including the mid-south region of upper Egypt. All experiments were carried out in accordance with ethical guidelines of Sohag and South Valley Universities, Egypt. Average milk production was 14.11±8.67 kg/day in the first farm and 26.41±2.11 kg/day in the second farm.

### Clinical examination and behavioral observation

Cows were observed for 30 min twice a day before milking in the morning and afternoon during their entrance to milking parlor according to focal sampling technique [19]. Breeding history was taken from records of farms. Post-partum estrous display, conception rate, and calving interval were recorded. Body condition score (BCS) was measured by an experienced examiner as previously described [20]. California mastitis test (bovine mastitis test, www.pbsanimalhealth.com) was performed, and the udder was examined in terms of teat elongation or deformity.

### Artificial rearing of calves

Calves were separated from their mother directly after birth. All calves were fed from their mother colostrum and milk during the 1^st^ week by milk bottles. After the 1^st^ week, calves were individually housed in pens (180 cm^2^ × 200 cm^2^) and offered artificial milk (10% of its body weight) twice a day from a bucket at 7:00 and 19:00. After the 3^rd^ week of age, solid feed composed of total mixed ration was provided *ad libitum*, and calves were housed in groups of seven each (pen area: 200 cm^2^ × 400 cm^2^). Weaning was performed when calves reached the body weight of 120 and 125 kg for female and male calves, respectively, irrespective of their age, and in this case, calves were called “gainer.” Calves that did not reach that body weight till 20-week-old were weaned at this age and called “non-gainer.”

### Statistical analysis

Data statistical analysis was performed by GraphPad Prism software. One-way ANOVA repeated measures was used for multiple comparisons, and t-test was used to compare the reproductive performance between the highest and lowest BCSs. The values were considered as significant only at p<0.05.

## Results and Discussion

### Effect of cross-sucking on calves

**Figure 1:**
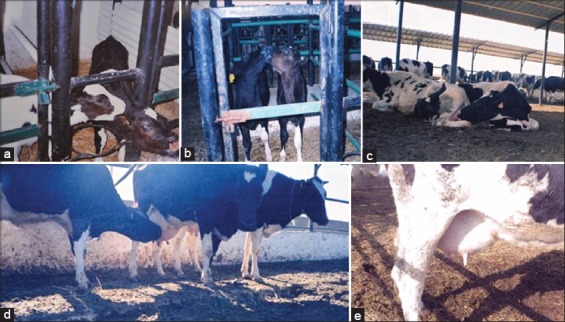
Photos from calves and cows involved in abnormal oral behavior. (a, b) Cross-sucking in calves after bucket feeding, (c) A cow engaged in self-sucking while the rest of the group was feeding), (d) A cow is sucking its neighbor, (e) teat elongation in a cow was involved in self- and cross-sucking.

**Figure 2:**
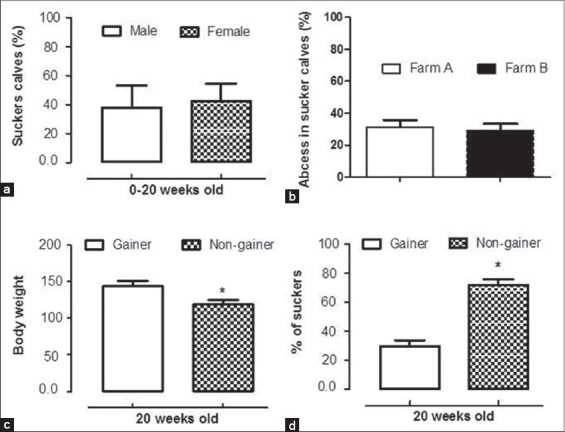
Cross-sucking in calves, (a) there were no significant differences between male and female calves in cross-sucking (t_(1,36)_=1.02, p=0.32), (b) the percentage of calves shown abscesses formations in ears and navels were not different between the two examined farms, (c) calves were divided according to their body weight at 20^th^ week into gainers and non-gainers, (d) percentage of suckers was calculated for gainers and non-gainers calves.

In applied animal ethology, behavioral observations are the standard methods for scientific studies. Incidences of cross-sucking in calves were shown in Figure-1a and b. No sex effect was found in calves involved in cross-sucking (Figure-2a). Abscess formation ranged from 32.4% to 39.1% in calves of either sex involved in cross-sucking. Calves displayed abnormal sucking developed abscesses in their ears and umbilicus that enhanced with age, reached their greatest extent at 6^th^ week of age, and then declined progressively to disappear before age of weaning. The number of calves affected with abscesses in ears and navel was negatively correlated with the age of calves (r=−0.41, p<0.05). Cross-sucking possibly is a form of non-nutritive sucking or tongue playing among calves (Figure-1a and b). Webb *et al*. [6] in their study on Holstein-Friesian calves concluded that tongue playing is a common form of non-nutritive oral behavior. The percentage of abscess formation in calves displayed cross-sucking from birth till waning was 34.40% and 32.23% in Farm A and B, respectively (Figure-2b). Despite the exhibition of cross-sucking among calves during the pre-weaning and post-weaning periods, abscesses of ears and navel reached the basal level before the age of weaning. The peak of abscess formation at either ears or navel was observed at the age of 6-week-old. This high percentage may result from injury or irritation caused by eruption of temporary incisor teeth in calves [21].

In this study, we showed that cross-sucking in calves was associated with reduced body weight at weaning (Figure-2d). The body weight of calves at the age of weaning is presented in Table-1. Furthermore, when calves were divided according to their body weight at 20^th^ week of age into gainers and non-gainers (Figure-2c), about 71% of non-gainers were involved in sucking (Figure-2d). In previous studies, cross-suckers suffered from abscesses at ears and navel, and bezoars (hair all formation) in the 4^th^ stomach was reported in veal calves at slaughterhouses [22]. If this abnormal behavior persisted in heifers, expressed as milk sucking in adulthood, culling is the necessary taken measure [14]. Some studies reported that increased milk and energy intake could reduce the incidences of non-nutritive sucking and cross-sucking [12,23-25]. Other studies reported that gradual weaning was found to reduce cross-sucking [15,26]. This study partially supports the hypothesis of insufficient oral satisfaction as an initiator of cross-sucking in calves that may persist and consolidate in the form of milk sucking in adulthood [27].

**Table-1:** Percentages of cross-sucking display, abscess fromation, and weaning weight in Holstein-Friesian calves.

Calves	Number		Cross-sucking (%)		Abscess (%) in suckers		Body weight at weaning	
			
	Male	Female	Male	Female	Male	Female	Male	Female
Farm A	112	107	38.08	42.61	34.13	34.40	122.11±17.2	122.03±16.5
Farm B	25	27	32.12	44.15	39.13	32.40	126.11±18.7	120.32±12.4

Data represent % or average of values for mean±SD. SD: Standard deviation

As it has been recently found, feeding milk to a calf (*ad-libitum* or up to 20% of its body weight) could reduce the signs of hunger and improve body weight before weaning [24,27]. Inter-sucking in heifers is related with teat injuries and secretion of a milky substance, and some farmers have reported that heifers start producing milk as a response of intense inter-sucking [28-30]. In addition, according to previous studies, if calves are fed milk from cows with mastitis and they suck the teats of other heifer calves, there is a risk that the bacteria is stored in the teats and udder till the day the heifer starts producing milk [31-33]. Since artificial milk feeding is generally used due to its ease and low cost, buckets with floating nipples could be used. However, extensive studies are required to study the effect of using this equipment in calf-hood period on animal welfare in terms of abnormal oral behaviors display. Although some treatments such as reduction of milk flow through the bucket and gradual weaning could reduce cross-sucking, sucking motivation is not completely satisfied [12,13].

### Effect of self- and inter-sucking on health and behavior of cows

**Figure 3:**
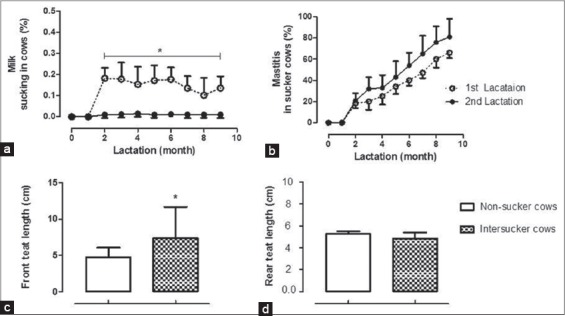
Inter-sucking in cows, (a) two-way ANOVA repeated measures revealed significant interaction of time by lactation period for the percentage of milk-sucking in cows (p<0.01), (b) among sucker cows, there were no significant differences in mastitis cases (p=0.61), (c) Student’s t-test in pairs revealed significant elongation of front teat in suckers compared to non-sucker cows (p<0.01), (d) there were no differences in rear teat length.

We examined inter-sucking in cows exhibited as self-sucking or sucking a neighbor (Figure-1c showing a cow engaged in self-sucking while the rest of the group was feeding and Figure-1d for cow is sucking its neighbor). The percentage of milk sucking (self- or inter-sucking) in dairy cows (Farm A and B as shown in Table-2). This percentage was also higher during the first compared to the second lactation period (Figure-3a). Two-way ANOVA repeated measures with Bonferoni *post-hoc* test indicated that mastitis incidences were not significantly influenced by the lactation period (Figure-3b). To exclude the influence of replacement procedures and culling that lead to underestimation of the standard error of data, we included missing values as previously referred by Greenland and Finkle [34]. It was notable that about 91% and 89% of sucker cows in Farm A and B, respectively, were observed displaying the behaviors of self-sucking and inter-sucking, whereas the rest exhibited only inter-sucking without self-sucking. Accordingly, the milk yield of sucker cows was reduced to about 80% of the average milk production. While cows that were sucking other cows without being sucked were free from mastitis and their milk production ranged from 14 to 20 kg/day.

**Table-2:** Percentages of milk sucking display, front teat elongation, post-partum estrus, and calving interval in Holstein-Friesian cows.

Dairy cows	Total number of cows	Milk sucking (%)	Front teats elongation (%) in sucker cows	Post-partum estrus in sucker cows	Calving interval in sucker cows
Farm A	1117	1.16	78.14	101.46±11.35	393.64±11.78
Farm B	1146	6.84	76.21	101.65±9.12	411.56±14.12

Data represent % or average of values for mean±SD. SD: Standard deviation

Results from California mastitis test in animals of both farms revealed that inter-sucking in cows led to increased mastitis incidences (about 54-53% mastitis in one quarter, 10-13% mastitis in 2 quarters, and 20-24% mastitis in 3 quarters) and elongation of the front teats. As a consequence of self- or inter-sucking in cows, there was elongation in front teats of sucker cows compared to that of non-sucked cows (Table-2; shown the percentage of front teat elongation). In sucked cows, front and not rear teat elongation (Figure-1e) could be possibly explained due to that fact that front is more easily accessible than the rear teats.

In addition, low BCS and interval to first service were negatively influenced since there is a genetic correlation between fertility (days to first service and non-return rate) and BCS. Moreover, cows that are thinner (lower BCS) have longer calving intervals [35,36]. Milk sucking reduces reproductive performance in terms of prolonged intervals to first post-partum estrus reaching to 3.5 months compared to about 40 days in normal cows, plus irregular estrous and increased the percentage of prolonged calving interval (Table-2 shows post-partum estrus and calving interval of sucker cows). This was in agreement with previous reports [20]. In addition, low BCS was observed in sucker cows; a fact that is correlated with a high incidence of repeat anestrus. It is also found that there is a high incidence of endometritis in cows with moderate and severe degrees of urovagina [37]. Furthermore, reduced reproductive performance in dairy cows with low BCS may be related to the acute angle of the tail (horizontal position of the vulva) that may result in the accumulation of urine and feces in the vulva, vagina, and endometritis [20].

The behavioral responses between suckers and non-sucker cows in regard to parlor entry and stockmen approaches are presented in Table-3. The results may refer to relatively high tension or anxiety during milking in terms of increased mean time of entry and dunging to the parlor. Approaching to stockmen and flight distance were also measured. Inter-sucker cows appeared to have greater values for mean entry time and dunging in parlor, longer flight distance, and lower values for approach to observer compared to non-suckers (Table-3). The obtained results may be related to signs of discomfort or pain as a result of subclinical mastitis.

**Table-3:** Behavioral differences between inter-sucker and non-sucker cows.

Dairy cows	Non-sucker cows	Inter-sucker cows	p value for t_(1, 21)_
Mean entry time to parlor (s/cow)	11.0±2.5^a^	16.0±4.0^b^	0.008
Dunging in the parlor (number/h)	3.0±0.4^a^	14.0±3.4^b^	0.003
Field flight distance (m)	0.5±0.1^a^	2.5±1.3^b^	0.043
Approach to observer (number/min)	12.2±1.5^a^	4.2±2.1^b^	0.025

Data represent average of values for mean±SD (n=11/group). a, b: Different superscripts indicate significant differences (p<0.05), SD: Standard deviation

The relationship between cross-sucking during calf-hood and milk sucking in adult cow is well illustrated in previous studies [16,27]. The present study also highlights the role of cross-sucking as a principal initiator of milk sucking in adult cows. This study emphasized the detrimental effects on animal health and performance. The resultant prolonged calving to conception interval may be due to later resumption of cyclic ovarian activity and fewer services per conception [20]. In contrast to the fact that culling due to inter-sucking was not common and the use of nose-rings as a control method was usually effective [30], the present study shows that the use of nose-ring with prongs was ineffective, further culling the affected animal was the ultimate choice for farm manger. Sucker cows had low BCS compared to non-suckers within the same age.

## Conclusion

The present study shows the impact of cross-sucking on the development of abscess at ears and navels, in addition with reduced body weight of calves at weaning. Furthermore, cows suffered from self- and inter-sucking had prolonged calving intervals, low BCS, and signs of discomfort behavior that may result from developed subclinical mastitis.

## Authors’ Contributions

MEM and AEA have formulated the research plan, conducted experimental procedures, and prepared the drafted manuscript. MEM and FAM prepared figures, tables, revised and submitted the manuscript. All authors read and approved the final manuscript.

## References

[1] Johnsen J F de, Passille A. M, Mejdell C. M, Bøe K. E, Grøndahl A. M, Beaver A, Rushen J, Weary D. M (2015). The effect of nursing on the cow - Calf bond. Appl. Anim. Behav. Sci.

[2] Khan M. A, Weary D. M, von Keyserlingk M.A.G (2011). Invited review: Effects of milk ration on solid feed intake, weaning, and performance in dairy heifers. J. Dairy Sci.

[3] Soberon F, Raffrenato E, Everett R. W, Van Amburgh M. E (2012). Preweaning milk replacer intake and effects on long-term productivity of dairy calves. J. Dairy Sci.

[4] David I, Bouvier F, Ricard E, Ruesche J, Weisbecker J. L (2014). Feeding behaviour of artificially reared Romane lambs. Animal.

[5] Wagner K, Barth K, Palme R, Futschik A, Waiblinger S (2012). Integration into the dairy cow herd: Long-term effects of mother contact during the first twelve weeks of life. Appl. Anim. Behav. Sci.

[6] Webb L E van, Reenen C. G, Berends H, Engel B de, Boer I. J, Gerrits W. J, Bokkers E. A (2015). The role of solid feed amount and composition and of milk replacer supply in veal calf welfare. J. Dairy Sci.

[7] Pempek J. A, Eastridge M. L, Botheras N. A, Croney C. C, Yoho W. S. B (2011). Effects of alternative housing and feeding systems on the behavior and performance of dairy heifer calves. Prof. Anim. Sci.

[8] Keil N. M, Langhans W (2001). Development of intersucking among dairy calves around weaning. Appl. Anim. Behav. Sci.

[9] Leruste H, Brscic M, Cozzi G, Kemp B, Wolthuis-Fillerup M, Lensink B. J, Bokkers E. A, van Reenen C. G (2014). Prevalence and potential influencing factors of non-nutritive oral behaviors of veal calves on commercial farms. J. Dairy Sci.

[10] Roth B. A, Barth K, Gygax L, Hillmann E (2009). Influence of artificial vs. mother-bonded rearing on sucking behavior, health and weight gain in calves. Appl. Anim. Behav. Sci.

[11] Roth B. A, Hillmann E, Stauffacher M, Keil N. M (2008). Improved weaning reduces cross-sucking and may improve weight gain in dairy calves. Appl. Anim. Behav. Sci.

[12] de Passillé A.M, Rushen J (2012). Adjusting the weaning age of calves fed by automated feeders according to individual intakes of solid feed. J. Dairy Sci.

[13] Fröberg S, Aspegren-Güldorff A, Olsson I, Marin B, Berg C, Hernández C, Galina C. S, Lidfors L, Svennersten-Sjaunja K (2007). Effect of restricted suckling on milk yield, milk composition and udder health in cows and behaviour and weight gain in calves, in dual-purpose cattle in the tropics. Trop. Anim. Health Prod.

[14] Appleby M. C, Weary D. M, Chua B (2001). Performance and feeding behaviour of calves on ad libitum milk from artificial teats. Appl. Anim. Behav. Sci.

[15] de Passillé A.M (2001). Sucking motivation and related problems in calves. Appl. Anim. Behav. Sci.

[16] Keil N. M, Audigé L, Langhans W (2000). Factors associated with intersucking in Swiss dairy heifers. Prev. Vet. Med.

[17] Svensson C, Jensen M. B (2007). Short communication: Identification of diseased calves by use of data from automatic milk feeders. J. Dairy Sci.

[18] Gransworthy P. C (2005). Calf and Heifer Rearing - Principles of rearing modern Dairy Heifer from Calve to Calving. Modern Calves and Heifers: Challenges for Rearing Systems.

[19] Mitlöhner F. M, Morrow-Tesch J. L, Wilson S. C, Dailey J. W, McGlone J. J (2001). Behavioral sampling techniques for feedlot cattle. J. Anim. Sci.

[20] Mahmoud M. E, Salman D (2015). Low body condition scoring as a detrimental factor to reproductive performance and behavior in dairy cattle and Seidi buffaloes. Assiut Vet. Med. J.

[21] Food Safety Inspection Service, (FSIS) (2013). Using Dentition to Age Cattle. U.S. Department of Agriculture. Washington, DC.

[22] Georg H, Ude G (2007). Reducing cross - Sucking of group housed calves by an environmental enriched building design. 9. Wissenschaftstagung Ökologischer Landbau. Beitrag archiviert unter.

[23] Jung L, Ledfors L (2001). The effect of mount of milk, milk flaw and the access to a rubber teat on cross-sucking and non-nutritive sucking in dairy calves. Appl. Anim. Behav. Sci.

[24] de Paula Vieira A, Guesdon V De, Passillé A.M Von, Keyserlingk M. A. G (2008). Behavioral indicators of hunger in dairy calves. Appl. Anim. Behav. Sci.

[25] Nielsen P. P, Jensen M. B, Ledfors L (2008). Milk allowance and weaning method affect the use of computer controlled milk feeder and development of cross-sucking in calves. Appl. Anim. Behav. Sci.

[26] Meale S. J, Leal L. N, Martín-Tereso J, Steelea M. A (2015). Delayed weaning of Holstein bull calves fed an elevated plane of nutrition impacts feed intake, growth and potential markers of gastrointestinal development. Anim. Feed Sci. Technol.

[27] Keil N. M, Audigé L, Langhans W (2001). Is intersucking in dairy cows the continuation of a habit developed in early life?. J. Dairy Sci.

[28] Jensen M. B (2006). Computer - Controlled milk feeding of group housed calves: The effect of milk allowance and weaning type. J. Dairy Sci.

[29] Rushen J de, Passillé A.N Von, Keyserlingk M. A. G (2008). The welfare of cattle. Introduction: What is Animal Welfare?. Springer, Dordrecht, Neitherland.

[30] Vaughan A, Miguel-Pacheco G G de, Passillé A.M, Rushen J (2016). Reciprocated cross sucking between dairy calves after weaning off milk does not appear to negatively affect udder health or production. J. Dairy Sci.

[31] De Vliegher S, Fox L. K, Piepers S, McDougall S, Barkema H. W (2012). Invited review: Mastitis in dairy heifers: Nature of the disease, potential impact, prevention, and control. J. Dairy Sci.

[32] Piepers S, Peeters K, Opsomer G, Barkema H. W, Frankena K De, Vliegher S (2011). Pathogen group specific risk factors at herd, heifer and quarter levels for intramammary infections in early lactating dairy heifers. Prev. Vet. Med.

[33] Loberg J, Lidfors L (2001). Effect of stage of lactation and breed on how dairy cows accept foster calves. Appl. Anim. Behav. Sci.

[34] Greenland S, Finkle W. D (1995). A critical look at methods for handling missing covariates in epidemiologic regression analyses. Am. J. Epidemiol.

[35] Dechow C. D, Rogers G. W, Clay J. S (2002). Heritability and correlations among body condition score loss, body condition score, production and reproductive performance. J. Dairy Sci.

[36] Kadarmideen H. N, Wegmann S (2003). Genetic parameters for body condition score and its relationship with type and production traits in Swiss Holsteins. J. Dairy Sci.

[37] Gautam G, Nakao T (2009). Prevalence of urovagina and its effects on reproductive performance in Holstein cows. Theriogenology.

